# Molecular Mechanisms Responsible for Mesenchymal Stem Cell-Based Modulation of Obstructive Sleep Apnea

**DOI:** 10.3390/ijms24043708

**Published:** 2023-02-13

**Authors:** Marija Zdravkovic, Carl Randall Harrell, Vladimir Jakovljevic, Valentin Djonov, Vladislav Volarevic

**Affiliations:** 1 Faculty of Medicine, University of Belgrade, Dr Subotica 8, 11000 Belgrade, Serbia; 2 Department of Cardiology, University Medical Center “Bežanijska Kosa”, Dr Zoza Matea bb, 11080 Belgrade, Serbia; 3 Regenerative Processing Plant, LLC, 34176 US Highway 19 N, Palm Harbor, FL 34684, USA; 4 Department of Physiology, Center of Excellence for Redox Balance Research in Cardiovascular and Metabolic Disorders, Faculty of Medical Sciences, University of Kragujevac, 69 Svetozar Markovic Street, 34000 Kragujevac, Serbia; 5 Department of Human Pathology, 1st Moscow State Medical, University IM Sechenov, Trubetskaya Street 8, Str. 2, 119991 Moscow, Russia; 6 Institute of Anatomy, University of Bern, Baltzerstrasse 2, 3012 Bern, Switzerland; 7 Department of Genetics, Faculty of Medical Sciences, University of Kragujevac, 69 Svetozar Markovic Street, 34000 Kragujevac, Serbia; 8 Department of Microbiology and Immunology, Faculty of Medical Sciences, University of Kragujevac, 69 Svetozar Markovic Street, 34000 Kragujevac, Serbia

**Keywords:** mesenchymal stem cells, obstructive sleep apnea, cell-based therapy, immunomodulation, neovascularization

## Abstract

Mesenchymal stem cells (MSCs) are adult stem cells that reside in almost all postnatal tissues where, due to the potent regenerative, pro-angiogenic and immunomodulatory properties, regulate tissue homeostasis. Obstructive sleep apnea (OSA) induces oxidative stress, inflammation and ischemia which recruit MSCs from their niches in inflamed and injured tissues. Through the activity of MSC-sourced anti-inflammatory and pro-angiogenic factors, MSCs reduce hypoxia, suppress inflammation, prevent fibrosis and enhance regeneration of damaged cells in OSA-injured tissues. The results obtained in large number of animal studies demonstrated therapeutic efficacy of MSCs in the attenuation of OSA-induced tissue injury and inflammation. Herewith, in this review article, we emphasized molecular mechanisms which are involved in MSC-based neo-vascularization and immunoregulation and we summarized current knowledge about MSC-dependent modulation of OSA-related pathologies.

## 1. Introduction

Obstructive sleep apnea (OSA) is a pathophysiological process characterized by repetitive interruptions of ventilation during sleep which occurs due to the partial or complete obstruction of the upper airways and results in increased respiratory effort [1,2]. The high magnitude of breathing efforts create strong negative intrathoracic pressure which negatively affects the heart function and may result in the development of left ventricular hypertrophy and heart failure. Apneic events are terminated by arousal, followed by increases in pulse and blood pressure and re-oxygenation which lead to the release of reactive oxygen species and inflammatory factors, leading to the development of oxidative stress and inflammation in affected tissues [1,2]. Additionally, repetitive breathing pauses lead to the derangements in gas exchange and oxygen desaturation which results in the activation of the sympathetic nervous system and disruption in normal hormone secretion. OSA-related increased catecholamine production contributes to the development of increased sympathetic tone and enhanced total peripheral resistance, resulting in the hypertension and tachycardia [1,2]. OSA-induced hypoxemia and decreased sleep time increase accumulation of aggregated amyloid-β in the brain of older patients and are considered important causes for the cognitive decline in the elderly [1]. Mid and long-term consequences of OSA-related pathological changes are excessive daytime sleepiness, insomnia, morning headaches, poorly controlled tachycardia and hypertension, irregular heartbeat, fatigue, memory deficits and apathy. Finally, long-lasting and uncontrolled OSA-induced oxidative stress, inflammation and ischemia may result in the development of cerebrovascular insult, heart failure and sudden death [1,2]. 

Mesenchymal stem cells (MSCs) are adult stem cells that reside in almost all postnatal tissues where, due to the potent regenerative, pro-angiogenic and immunomodulatory properties, regulate tissue homeostasis [3]. Alarmins and inflammatory cytokines, released from injured parenchymal and immune cells, increase synthesis of growth factors, angiomodulatory and immunoregulatory proteins in MSCs, altering their phenotype and function [4]. In addition, chemokines, which are elevated in inflamed tissues, induce enhance expression of chemokine receptors and integrins on MSCs, enabling recruitment of MSCs from distinct tissues at the site of injury and inflammation [5]. In line with these findings, OSA-triggered intermittent hypoxia, oxidative stress and inflammation induce early and massive release of MSCs from their niches into circulating blood [6]. Recruited MSCs migrate in ischemic and inflamed tissue where, by suppressing inflammatory immune cells and by promoting neo-angiogenesis and re-oxygenation, participate in the attenuation of OSA-related pathological changes [7,8]. Herewith, in this review article, we emphasized molecular mechanisms which are involved in MSC-based neo-vascularization and immunoregulation and we summarized current knowledge about MSC-dependent modulation of OSA-related pathologies. An extensive literature review was carried out in November 2022 across several databases (MEDLINE, EMBASE, Google Scholar), from 1990 to present. Keywords used in the selection were: “mesenchymal stem cells”, “obstructive sleep apnea”, “therapy”, “neovascularization”, “regeneration”, “immunomodulation”. All journals were considered and, initial search retrieved 147 articles. The abstracts of all these articles were subsequently reviewed by three of the authors (MZ, CRH and VV) independently to check their relevance to the subject of this manuscript. Eligible studies had to delineate molecular and cellular mechanisms responsible for the MSC-based attenuation of OSA-related pathological changes and their findings were analyzed in this review.

## 2. Molecular Mechanisms Responsible MSC-Dependent Attenuation of OSA-Induced Ischemia

MSCs are angiomodulatory stem cells that in paracrine manner, through the activity of MSC-derived pro-angiogenic factors, induce generation of new blood vessels from a pre-existing vascular plexus [9]. Stimulatory effects of MSC-sourced growth factors and angioregulatory proteins on survival and proliferation of endothelial cells (ECs) are mainly responsible for MSC-dependent neovascularization and re-oxygenation of OSA-related ischemic tissues [9,10]. OSA-induced hypoxemia enhances production of several pro-angiogenic factors in MSCs, including vascular endothelial growth factor (VEGF), placental growth factor (PLGF), angiopoietin-1 (Ang1), interleukin (IL)-6, platelet-derived endothelial cell growth factor (PD-ECGF), stromal derived factor 1 (SDF-1), hepatocyte growth factor (HGF) [7,11]. These MSC-sourced angiomodulatory factors activate various intracellular signaling pathways in ECs enhancing their survival, viability and proliferation [10,11].

An interaction between MSC-derived VEGF and VEGFR2 of ECs recruits several adapter molecules in ECs, including SH2 domain containing adaptor protein B (SHB), SRC proto-oncogene (SRC), SH2 domain containing 2 A (TSAD), fibroblast growth factor receptor substrate 2 (FRS2) [12]. The most important pathway of VEGF-dependent regulation of EC proliferation involves phosphoinositide phospholipase C (PLCγ)-mediated activation of protein kinase C (PKC) and downstream induction of the extracellular signal-regulated kinase (ERK) which results in the suppression of several pro-apoptotic molecules in ECs [13]. Additionally, VEGF:VEGFR2-mediated activation of phosphoinositide 3-kinase (PI3K)-AKT signaling enhances survival of ECs since AKT modulates mTOR activity, inhibits caspase-dependent apoptosis and promotes cyclinD1 activity in Nuclear factor-κB (NF-κB) -dependent manner [12,13].

MSC-derived PLGF has high amino acid homology with VEGF [14]. It binds to VEGFR-1 and enhances the pro-angiogenic effects of VEGF by enhancing survival and growth of ECs [14]. OSA-triggered hypoxemia induces increased production of PLGF in MSCs that reside in their niches within ischemic tissues [7,8]. Massive production of PLGF increases its concentration in the blood which, in turn, enhances mobilization of bone marrow-derived MSCs (BM-MSCs) [15]. Accordingly, OSA induced massive release of MSCs from the bone marrow in the peripheral blood of experimental rats [6]. Significantly higher number of BM-MSCs were observed in the systemic circulation of the rats subjected to recurrent OSA than in the blood of control, healthy animals [6].

MSC-sourced Ang1 is very potent anti-apoptotic factor that promotes survival of ECs [9]. Ang1 induces phosphorylation of Tie2 receptor and activates PI3K in dose-dependent manner [16]. Ang1/Ti2/PI3K signaling pathway elicits AKT-dependent modulation of mTOR activity and prevents caspase-driven apoptosis of ECs [9,10,16]. 

Han et al. showed that MSC-derived IL-6 and PD-ECGF could induce increased proliferation of ECs [17]. Upon binding to its receptors gp80 and gp130, IL-6 increases EC proliferation via the activation of the Janus kinase (JAK)/signal transducer and activator of transcription (STAT) and mitogen-activated protein kinase (MAPK) pathways [9,17]. MSC-sourced PD-ECGF is thymidine phosphorylase which catalyzes the reversible phosphorylation of thymidine to deoxyribose-1-phosphate and thymine, regulating thymidine homeostasis. By increasing intranuclear concentration of thymin, MSC-derived PD-ECGF enhances DNA synthesis and induces increased proliferation of ECs [9,17]. 

Chang et al. and Wang et al. suggested that MSC-derived HGF and SDF-1 could improve migratory properties of ECs and their progenitors, enhancing wound healing of injured blood vessels [18,19]. HGF exerts its pro-angiogenic activity through tyrosine phosphorylation of its specific receptor, c-Met, which is expressed on ECs of newly formed blood vessels [18]. MSC-derived HGF is responsible for MSC-dependent wound healing and restoration of blood vessels’ integrity in injured tissues [9,17,18]. Once MSC-derived HGF activates c-Met, several kinases (ERK, c-Jun N-terminal kinases (JNK), PI3K and p38MAPK) become phosphorylated in ECs, resulting in their enhanced proliferation and migration which finally restores integrity of injured blood vessels [18]. MSCs exposed to OSA-induced hypoxemia produce large amount of HGF and have increased capacity for wound healing [7,8,20]. When compared with control serum, apneic serum significantly increased MSC-dependent endothelial wound healing and restoration of blood vessel integrity [20]. 

Wang et al. showed that SDF-1, secreted by MSCs, regulated migratory properties of endothelial progenitor cells (EPCs) and enhanced neo-angiogenesis and wound healing in ischemic tissues by stimulating tube formation [19]. By binding to CXCR4 on EPCs, MSC-sourced SDF-1 enhances migration and recruitment of EPCs to the sites of ischemic injury, crucially contributing to the neovascularization [19]. CXCR4 is expressed on the membrane of MSCs and is crucially responsible for optimal MSCs homing to the site of injury and inflammation [21]. In line with these findings are results obtained by Carreras and colleagues who showed that apneic serum significantly increased migratory properties and chemotaxis of CXCR4-expressing MSCs [20]. Adhesion of MSCs to ECs was significantly greater when these cells were exposed to apneic serum since OSA-triggered hypoxemia induced cytoskeletal remodeling that resulted in over-expression of integrins and chemokine receptors on the membrane of MSCs, enabling firm adhesion and optimal MSCs:ECs cross-talk in ischemic tissues [20]. 

## 3. MSC-Dependent Suppression of OSA-Triggered Inflammation

OSA-related recurrent hypoxia triggered early inflammatory response manifested by the elevation of pro-inflammatory cytokines in systemic circulation and affected tissues [22,23]. Significantly higher level of IL-1β was observed in serum samples of experimental rats that were subjected to the recurrent apneas, compared with healthy animals [24]. IL-1β is major inflammatory cytokine mostly released by activated macrophages, dendritic cells (DCs) and neutrophils [23]. In injured and inflamed tissue, increased influx of inflammatory cells in affected tissues and immune cell-driven inflammatory cascade are initiated upon the binding of IL-1β to its cognate receptor, IL-1RI on the membrane of ECs [23]. IL-1β-dependent activation of IL-1RI recruits IL-1 receptor accessory protein (IL-1RAP) to the membrane of ECs, resulting in the creation of a trimeric complex which activates MyD88-dependent intracellular cascade [23]. MyD88 links IL-1R ternary complex with IL-1R-associated kinases (IRAK) and MAPK, resulting in the activation of several transcriptional factors (NF-κB, activator protein 1 (AP-1)) that increase expression of genes responsible for the production of E and P selectins and integrin ligands [23]. Accordingly, by inducing increased concentration of IL-1β in affected tissues, OSA-triggered hypoxia enhances expression of adhesion molecules (selectins and integrin ligands) on ECs enabling massive influx of immune cells and consequent development of inflammation [22,23]. Furthermore, in tissue resident macrophages, activation of IL-1β/IL-1RI/IL-1RAP-driven inflammatory cascade elicit increased production of inflammatory chemokines and cytokines (including tumor necrosis factor alpha (TNF-α) that acts synergistically with IL-1β during the early phase of inflammation), enabling massive recruitment of circulating leukocytes in ischemic and injured tissues [22,23]. 

Considering the fact that IL-1β is very important cytokine in inflammatory response, suppression of its biological effects effectively protects tissues from inflammation-induced injuries [25]. Interleukin 1 receptor antagonist (IL-1Ra) is a naturally occurring cytokine which acts as an inhibitor of IL-1β [25]. A well-characterized subpopulation of IL-1Ra-expressing MSCs have been described in injured lungs of experimental animals subjected to hypoxia and inflammation [26]. MSCs which are exposed to hypoxic conditions and inflammatory microenvironment produce large amount of immunosuppressive IL-1Ra, as a response to the on-going inflammation [25,26]. When MSC-derived IL-1Ra binds to the IL-1RI on ECs, binding of IL-1β is blocked, IL-1β:IL-1RI:IL-1RAP trimeric complex is not formed and pro-inflammatory MyD88/IRAK/MAPK-driven signaling is not elicited [25]. Accordingly, various pro-inflammatory events initiated by IL-1β:IL-1RI signaling, including the over-expression of adhesion molecules on ECs, increased synthesis and release of inflammatory chemokines and cytokines accompanied with massive recruitment of neutrophils, monocytes, and lymphocytes, are inhibited by MSC-sourced IL-1Ra [25]. In line with these findings, results obtained in animal model of OSA induced by recurrent airway occlusions, showed that MSCs efficiently suppressed systemic inflammatory response by attenuating IL-1β-driven inflammation [24]. The rats subjected to recurrent obstructive apneas (60 per hour, lasting 15 s each, for 5 h) were randomly divided in the two experimental groups to intravenously received saline or BM-MSCs (5 × 10^6^ cells) which were infused 30 min before the application of airway obstruction [24]. Concentration of IL-1β was significantly lower in the serum samples of BM-MSC-treated apneic rats than in MSC-untreated apneic animals, indicating that intravenously injected MSCs efficiently suppressed OSA-induced IL-1β-driven systemic inflammation [24]. 

Interestingly, down-regulated level of IL-1β was accompanied with elevated concentration of immunosuppressive IL-10 in the serum samples of BM-MSC-treated apneic rats [24]. MSC-derived IL-10 is very potent anti-inflammatory and immunoregulatory cytokine that was mainly responsible for MSC-dependent suppression of various autoimmune and inflammatory diseases [27]. MSC-sourced IL-10 alters phenotype and function of inflammatory DCs, T lymphocytes and macrophages, immune cells that are responsible for the development of OSA-related pathological changes in the inflamed tissues [7,27]. Through the production of IL-10, MSCs inhibit maturation of DCs and suppress activation and proliferation of T cells [27,28]. MSC-derived IL-10 induce generation of tolerogenic, immuno-suppressive phenotype in DCs. These tolerogenic DCs have immature phenotype, do not optimally produce pro-Th1 and pro-Th17 inflammatory cytokines (TNF-α, IL-12, IL-1β, IL-23) and therefore, are not capable to induce activation of naïve CD4+ and CD8+ T cells and are incapable to generate inflammatory Th1 and Th17 cells [27]. Additionally, MSCs, in IL-10-dependent manner, induce alternative activation of macrophages [27,28]. In alternatively activated macrophages, MSC-derived IL-10 inhibits synthesis of inflammatory cytokines (IL-1β, TNF-α) and enhances synthesis of immunosuppressive transforming growth factor beta (TGF-β) which inhibits activation of JAK-STAT signaling pathway, induces the G1 cell cycle arrest and attenuates expansion of activated T lymphocytes [27,28]. Therefore, MSCs, in paracrine manner, through the anti-inflammatory effects of MSC-sourced IL-1Ra and IL-10, inhibit IL-1β-driven systemic inflammatory response, generate immunosuppressive phenotype in immune cells and create immunosuppressive microenvironment in ischemic and inflamed tissues of apneic animals, enabling restoration of tissue homeostasis [7,24,27]. 

## 4. Therapeutic Potential of MSCs in the Treatment of Chronic Obstructive Pulmonary Disease (COPD)-OSA Overlap Syndrome

MSCs are multipotent stem cells that spontaneously differentiate in the cells of mesodermal origin (adipocytes, chondrocytes and osteocytes) [29]. However, large number of experimental studies demonstrated that MSCs which are cultured under specific conditions, may differentiate in the cells of endothelial lineage, as well [29,30,31]. MSCs which were exposed to the 2% fetal calf serum (FCS) and 2% VEGF (50 ng/mL) for 7 days, differentiated in the functional ECs [30,31]. Very low expression of MSC-specific markers (CD105, CD73, CD166, CD90, and CD44) and high expression of EC-specific markers (kinase insert domain receptor (KDR), vascular endothelial growth factor receptor 1 (VEGFR1)), vascular cell adhesion protein 1 (VCAM-1), von Willebrand factor) were observed on ECs that differentiated from VEGF-treated MSCs [30,31]. Importantly, Weibel-Palade bodies and tube-like capillary structures were visible in the semisolid medium of VEGF-treated MSCs, indicating that newly generated ECs were functional, vasculature-forming cells capable to induce neo-angiogenesis in ischemic and injured tissues [30,31,32]. 

In line with these findings are results obtained by Bi et al. and Chen et al. who investigated therapeutic potential of MSCs in animal model of chronic obstructive pulmonary disease (COPD)-OSA overlap syndrome (OS) (Table 1). They demonstrated that MSCs differentiated in functional ECs and efficiently attenuated OS-triggered vascular injury in the lungs [33,34]. OS was induced by exposing experimental rats to the cigarette smoke ((15 cigarettes at once, two times daily) and intermittent hypoxia (with ~99% nitrogen (N_2_) for 30 s and air (~21% O_2_) every day for 90 s, 30 times per hour, 8 h per day) for 8 weeks [33,34]. OS rats were, 24 h after cigarette smoke and intermittent hypoxia exposure, randomly divided in experimental groups to intravenously receive either saline or BM-MSCs (2 × 10^6^ cells; one time/week, for 4 weeks) [33,34]. It is well known that injured parenchymal cells release alarmins that attract exogenously administered MSCs to the site of injury [21]. Accordingly, several weeks after their systemic infusion, BM-MSCs were detected in the lungs and aortas which were the most severely injured by cigarette smoke and hypoxia [33,34]. 

BM-MSC reduced emphysematous changes and improved respiratory function of OS rats [34]. Significantly enlarged alveolar spaces, fractured alveolar septums, swollen bronchial epithelial cells, narrowed bronchial tubes, thickened alveolar walls, fused alveoli, large number of cystic cavities and inflammatory cell infiltrates which were observed in the bronchial and lung tissues of saline-treated OS rats, were not seen in OS-affected lungs of BM-MSCs-treated animals [34]. Majority of intravenously injected BM-MSCs were trapped in OS-injured lungs [34]. Beneficial effects of BM-MSCs were relied on their capacity to generate new blood vessels, to inhibit influx of inflammatory immune cells and to suppress oxidative stress in OS-injured lungs [34]. Mean alveolar number was significantly higher in BM-MSC-treated than in saline-treated OS rats, confirming beneficial effects of BM-MSCs in the alleviation of OS-triggered lung injury [34].

Similarly, BM-MSCs efficiently reduced pathological changes in the aortas of saline-treated OS rats [33]. Cigarette smoke and intermittent hypoxia induced severe damage in the aortas’ vascular walls of saline-treated OS rats [33]. Boundary between the outer membrane, middle membrane and intima was not clear. The elastic membrane of the media was not visible. Smooth muscle cells and ECs were disordered and swollen [33]. Importantly, these OS-triggered pathological changes were not seen in the aortas of OS rats that received BM-MSCs [33]. Preserved vascular wall structure, thickened tunica media, proliferative smooth muscle cells and slightly swollen ECs were observed in the aortas of BM-MSC-treated OS rats [33]. BM-MSCs inhibited apoptosis of ECs and modulated expression of endotheliocyte injury-related genes in the aortas [33]. BM-MSCs increased expression of endothelial nitric oxide synthase (eNOS) gene and down-regulated expression of endothelin (ET)-1 and VCAM-1 genes [33]. ET-1 negatively regulates the release of nitrixc oxide (NO) in the endothelium while VCAM-1 enhances massive recruitment of circulating leukocytes at the site of damage [33]. Therefore, by suppressing ET-1 and VCAM-1 expression in ECs, BM-MSCs enabled vasodilatation, re-oxygenation and inhibited influx of inflammatory immune cells in injured aortas of OS rats, crucially contributing to their enhanced repair and regeneration [33].

Importantly, all OS-related clinical signs and symptom were significantly attenuated in BM-MSCs-treated OS rats. Abnormal breathing, audible wheezing, intermittent cough, intense salivation, yellow skin, loss of appetite and loss of weight which were observed in saline-treated OS rats, were not seen in BM-MSC-treated OS animals. Physiological breathing, normal loco-motor activity, slightly decreased appetite without significant change in body weight, observed in BM-MSC-treated OS rats, confirmed beneficial effects of intravenously infused BM-MSCs in the attenuation of OS-induced pathological changes.

## 5. Molecular Mechanisms Responsible for the Attenuation of OSA-Induced Fibrosis

In addition to vascular injury, long-term exposition to cigarette smoke and hypoxia resulted in increased accumulation of collagen fibers in the outer and medial membrane of aortic and atrial tissues in saline-treated OSA rats [35,36]. Recurrent apneas induces severe pathological changes in the morphology and structure of descending aortas [35]. Significantly increased wall thickness, enlarged lumen diameter, many ruptured elastin fibers and massive deposition of collagen fibers in the tunica media were observed in aortas of saline-treated OSA rats and resulted in the development of aortic hypertrophy [35]. Significantly increased levels of the NADPH oxidase, decreased expression of eNOS and inducible nitric oxide synthase (iNOS) genes, increased activity of angiotensin-converting enzyme (ACE)-1 were noticed in saline-treated OSA rats, indicating that OSA-induced oxidative stress and dysfunction of renin-angiotensin system were mainly responsible for aortic remodeling [35]. BM-MSCs (5 × 10^6^ cells), which were intravenously infused 24 h after OSA induction and every 4 days for three weeks, prevented collagen deposition, normalized vascular remodeling and completely reversed aortic structural changes by attenuating superoxide anion production, by suppressing ACE-1 activity and by increasing iNOS expression in the aortas of OS rats [35].

In line with these findings are results obtained by Ramos and colleagues who demonstrated beneficial effects of MSCs in the attenuation of OSA-induced atrial fibrillation in experimental rats [36]. Recurrent airway obstructions promoted myocardial inflammation which was followed by fibrosis [36]. OSA generated potent inflammatory response which was manifested by significant increase in plasma levels of IL-1β [36]. Systemic inflammation resulted in the massive, diffuse and homogenous collagen deposition in the atrial walls of OSA rats [36]. Interestingly, significantly increased accumulation of collagen fibers was observed only in atrial and not in ventricular walls and resulted in the development of atrial fibrillation (AF) [36]. Significantly decreased expression of matrix metalloproteinase (MMP)-2 was noticed in atrial walls of OSA rats compared to healthy animals, suggesting that reduced activity of MMP-2 collagen degrading enzyme was mainly responsible for the development of OSA-induced atrial structural remodeling and fibrosis [36].

It is well known that MSCs, in MMP-2 dependent manner, regulate collagen synthesis and in IL-1Ra-dependent manner suppress IL-1β driven inflammation [25,37]. Accordingly, significantly decreased plasma levels of inflammatory IL-1β, increased MMP-2 activity and reduced accumulation of collagen fibers were observed in the atrial walls of BM-MSC-treated OSA rats [36]. Importantly, MSC-dependent suppression of atrial fibrosis resulted in the attenuation of AF and led to the significant improvement of cardiac function in OSA rats [36], suggesting therapeutic potential of MSCs in the treatment of OSA-induced fibrosis and tissue remodeling.

## 6. Conclusions

**Figure 1:**
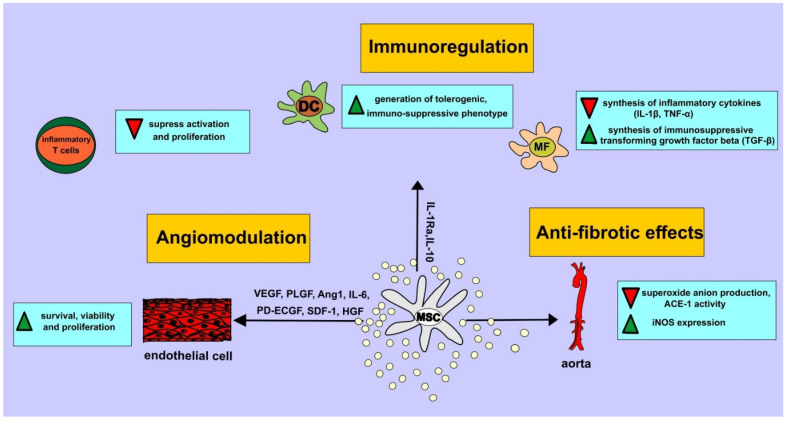
Immunoregulatory, angiomodulatory and anti-fibrotic effects of MSC-sourced factors in OSA-injured and inflamed tissues. MSCs in paracrine manner attenuate OSA-induced ischemia, inflammation and fibrosis. MSC-derived angiomodulatory factors (vascular endothelial growth factor (VEGF), placental growth factor (PLGF), angiopoietin-1 (Ang1), interleukin (IL)-6, platelet-derived endothelial cell growth factor (PD-ECGF), stromal derived factor 1 (SDF-1), hepatocyte growth factor (HGF)) activates various intracellular signaling pathways in endothelial cells (ECs) enhancing their survival, viability and proliferation. MSCs, in paracrine manner, through the anti-inflammatory effects of MSC-sourced IL-1Ra and IL-10, inhibit IL-1β-driven systemic inflammatory response, generate immunosuppressive phenotype in immune cells and create immunosuppressive microenvironment in ischemic and inflamed tissues. MSCs prevented collagen deposition, normalized vascular remodeling and completely reversed aortic structural fibrotic changes by attenuating superoxide anion production, by suppressing ACE-1 activity and by increasing iNOS expression in the aortal tissue.

The results obtained in animal studies demonstrated that MSCs, due to their potent regenerative, angiomodulatory and immunoregulatory properties, efficiently attenuated OSA-induced vascular injuries, inflammation and fibrosis (Table 2) [7,8,20,24,33,34,35,36]. Through the activity of MSC-sourced anti-inflammatory and pro-angiogenic factors, MSCs reduce hypoxia, suppress inflammation, prevent fibrosis and enhance regeneration of damaged cells in OSA-injured tissues (Figure 1) [7,8,20,24,33,34,35,36]. However, it should be emphasized that beneficial effects of MSCs in the attenuation of OSA-related pathological changes should be confirmed in clinical studies before MSCs could be offered as new remedies in OSA treatment.

## Figures and Tables

**Table 1:** Molecular mechanisms responsible for the beneficial effects of MSCs in the treatment of COPD-OSA overlap syndrome.

Tissue Source of MSC	Route of Injection	Total Number of MSC	Target Tissue	Mechanism of Action	Beneficial Effect(s)	Ref. No.
bone marrow	intravenous	2 × 10^6^	aorta	modulated expression of apoptosis-related genes in ECs	inhibition of apoptosis of ECs	[33]
bone marrow	intravenous	2 × 10^6^	aorta	increased expression of eNOS gene and down-regulated expression of ET-1 gene in ECs	increased synthesis of NO; enhanced vasodilatation	[33]
bone marrow	intravenous	2 × 10^6^	aorta	down-regulated expression of vascular cell adhesion protein 1 (VCAM-1) gene in ECs	reduced influx of inflammatory cells and attenuated inflammation	[33]
bone marrow	intravenous	2 × 10^6^	lungs	differentiation in functional CD34-expressing ECs	generation of new blood vessels and re-oxygenation	[34]
bone marrow	intravenous	2 × 10^6^	lungs	decreased malondialdehyde and increased superoxide dismutase activity	prevention of oxidative stress-induced injury of alveolar epithelial cells	[34]
bone marrow	intravenous	2 × 10^6^	lungs	suppressed influx of inflammatory cells	reduced number of lung-infiltrated immune cells and attenuated inflammation	[34]

Abbreviations: mesenchymal stem cells (MSCs), chronic obstructive pulmonary disease (COPD); obstructive sleep apnea (OSA); endothelial cells (ECs); endothelial nitric oxide synthase (eNOS); endothelin (ET)-1; nitric oxide (NO); vascular cell adhesion protein 1 (VCAM-1).

**Table 2:** Therapeutic potential of MSCs in the treatment of obstructive sleep apnea.

OSA-Related Pathological Condition	MSC-Derived Factor(s)	Mechanism of Action	Beneficial Effect(s)	Ref. No.
OSA-induced ischemia	HGF	increased proliferation of ECs; generation of new blood vessels	increased endothelial wound healing; restoration of blood vessel integrity	[20]
OSA-induced inflammation	IL-1Ra; IL-10	inhibited recruitment of inflammatory immune cells in injured tissues; enhanced alternative activation of macrophages; attenuated proliferation n of inflammatory T cells; increased expansion of immunosuppressive Tregs	generation of immunosuppressive microenvironment; enhanced repair and regeneration of injured tissues; restoration of tissue homeostasis	[27,28]
COPD-OSA overlap syndrome	NO	enhanced vasodilatation; re-oxygenation of ischemic tissues	improved breathing, loco-motor activity and appetite	[33,34]
OSA-induced fibrosis	NO; IL-10	inhibited collagen deposition; suppressed ACE-1 activity; increasing iNOS expression; attenuated superoxide anion production	normalized vascular remodeling; completely reversed aortic structural changes	[35]
OSA-induced atrial fibrillation	MMP-2 IL-1Ra	reduced accumulation of collagen fibers in atrial walls; inhibition of IL-1β driven inflammation	reduced atrial fibrosis; improved cardiac function	[36]

Abbreviations: mesenchymal stem cells (MSCs), chronic obstructive pulmonary disease (COPD); obstructive sleep apnea (OSA); hepatocyte growth factor (HGF); interleukin 1 receptor antagonist (IL-1Ra); nitric oxide (NO); interleukin (IL); matrix metalloproteinase (MMP); T regulatory cells (Tregs); angiotensin-converting enzyme (ACE-1); inducible nitric oxide synthase (iNOS); endothelial cells (ECs).

## Data Availability

Not applicable.
